# Expulsion of iron-rich ferritin via CD63-mediated exosome drives ferroptosis resistance in ovarian cancer cells

**DOI:** 10.3389/fcell.2025.1532097

**Published:** 2025-03-10

**Authors:** Anna Martina Battaglia, Alessandro Sacco, Emanuele Giorgio, Lavinia Petriaggi, Julia Elzanowska, Ana Rita Cruz, Luis Rocha, Catarina Esteves Pereira, Maria Carolina Strano Moraes, Luca Palazzo, Claudia De Vitis, Bruno Costa-Silva, Flavia Biamonte

**Affiliations:** ^1^ Laboratory of Biochemistry and Cell Biology, Department of Experimental and Clinical Medicine, Magna Graecia University of Catanzaro, Catanzaro, Italy; ^2^ Systems Oncology Laboratory, Champalimaud Foundation, Lisbon, Portugal; ^3^ Department of Molecular Medicine and Medical Biotechnology, University of Naples “Federico II”, Naples, Italy; ^4^ Department of Clinical and Molecular Medicine, Sant’ Andrea Hospital-Sapienza University of Rome, Rome, Italy

**Keywords:** ovarian cancer, ferroptosis resistance, exosome, ferritin, iron

## Abstract

**Introduction:**

Ferroptosis is a promising new target for ovarian cancer (OVCA) treatment. However, some OVCA cell types resist the induction of ferroptosis by limiting the intracellular accumulation of the labile iron pool (LIP).

**Methods:**

HEY, COV318 and PEO4 were treated with erastin and assessed for cell viability by using PI flow cytometry assays. Erastin-affected iron metabolism was analysed by using FerroOrange assay, Western Blot (WB) analysis of ferritin heavy chain (FtH), transferrin receptor (CD71), and ferroportin (FPN). Mitochondrial reactive oxygen species (mitROS) and lipid peroxidation were quantified via MitoSOX and BODIPY-C11 flow cytometry assays, respectively. Exosomes (EVs) were collected from cell culture media through ultracentrifugation and then enumerated and analyzed by Nanoparticale Tracking Analysis (NTA) and transmission electron microscopy (TEM). CD63 protein expression in EVs was measured through WB by using CD9 as a loading control. Loss-of-function assays for FtH and CD63 were performed by using siRNA-mediated transient transfection.

**Results:**

We demonstrate that erastin treatment (8 µM, 8 h) is accompanied by the release of iron-rich ferritin via EV pathway in COV318 and PEO4 OVCA cells, thus failing to exert cytotoxic effects. Mechanistically, erastin causes the upregulation of CD63, a tetraspanin involved in forming multivesicular bodies (MVBs) and EVs, and the increase of MBVs assessed by transmission electron microscopy. Consistent with these findings, EV isolation followed by nanoparticle tracking analysis revealed a significant increase in EVs/cell in erastin-treated COV318 and PEO4 cells. Notably, EVs harvested from these cells contained CD63 and FtH, a major iron-storage protein. Inhibition of EV biogenesis with GW4869 prevented FtH release and restored LIP accumulation, lipid peroxidation, and ferroptosis sensitivity in COV318 and PEO4 cells.

**Discussion:**

Overall, our results indicate that OVCA cells can utilize CD63+ EVs to secrete iron-rich ferritin as a mechanism to evade erastin-induced ferroptosis. These findings suggest that combining erastin with EV inhibitors could offer promising strategy for overcoming ferroptosis resistance in OVCA.

## 1 Introduction

Over the past decade, ferroptosis has garnered significant attention as a novel therapeutic strategy to target tumor cells (Dixon et al., 2012; Dixon and Stockwell, 2019; Stockwell, 2022). Ferroptosis is a regulated form of cell death (RCD) driven by iron-dependent lipid peroxidation. When iron accumulates within the cell as redox-active labile iron pool (LIP), it can react with hydrogen peroxide (H_2_O_2_) via Fenton reactions, producing reactive oxygen species (ROS) such as hydroxyl radical (^.^OH) and superoxide radical (O_2_^−^). In the presence of oxidizable lipids, such as polyunsaturated phospholipids (PUFA-PLs), and in the absence of sufficient antioxidant defenses, particularly those mediated by glutathione peroxidase 4 (GPX4), ROS can induce the peroxidation of both organelle and plasma membranes and ultimately leading to ferroptotic cell death. Additionally, iron and its derivatives, including heme and iron-sulfur [Fe-S] clusters, are critical for the activity of enzymes such as NADPH oxidases (NOXs) and lipoxygenases (LOXs), which contribute to ROS production or are involved in lipid peroxidation (Battaglia et al., 2020). Recently, a growing number of ferroptosis inducers (FINs) have been identified. Mechanistically, FINs are typically classified into the following categories: i) Class I, which inhibits cystine import via the System Xc^−^ (e.g., erastin, sorafenib, artesunate) and, consequently, depletes glutathione (GSH); ii) Class II and III, which directly or indirectly inhibit GPX4 and consume endogenous membrane antioxidant CoQ10 (e.g., RSL3 and FIN56); iii) Class IV, which induces lipid peroxidation by increasing LIP levels or oxidizing iron (Hassannia et al., 2019; Nie et al., 2022; Zhang et al., 2022). Concerning the Class IV FINs, the phenomenon of “iron addiction”, first described by Basuli et al. as the tendency of certain cancer cells to accumulate elevated iron levels by enhancing iron intake or limiting iron efflux, has led to the identification of a new class of compounds, such as FAC and ferlixit, which promote intracellular iron overload and enhance ferroptotic cell death (Basuli et al., 2017; Lelièvre et al., 2020; Guo et al., 2021; Battaglia et al., 2022; 2023b). Although FINs hold promise as potent antitumor agents, certain cancer cells exhibit resistance to ferroptosis due to inherited genetic determinants and/or metabolic adaptations. Furthermore, cancer cells can develop resistance to ferroptosis overtime through a variety of genetic, epigenetic, and metabolic changes (Lei et al., 2022; Zhang et al., 2022). Recently, we and others have demonstrated that cancer cells may reprogram iron metabolism in response to ferroptosis-inducing stimuli, maintaining low intracellular LIP levels to evade cell death (Battaglia et al., 2022). In line with this concept, experimental data suggest that the use of iron-chelating agents or the targeting of genes involved in intracellular iron homeostasis can prevent ferroptotic cell death by reducing the pool of redox-active iron (Chen X. et al., 2020). For instance, inhibition of the serine/threonine kinase ATM, which is normally activated in response to DNA damage, protects cells from ferroptosis by promoting iron storage, through the overexpression of ferritin heavy (*FtH*) and light chains (*FtL*), as well as iron export, via the overexpression of ferroportin (*FPN*) (Chen P. H. et al., 2020). Additionally, activation of nuclear factor erythroid 2-related factor 2 (NRF2) pathway limits ferroptosis by enhancing *FtH* transcription (Yan et al., 2023). More recently, Brown CW. et al., demonstrated that the export of iron-rich ferritin via the multivesicular bodies/exosomes (MVBs/EVs) pathway protects breast cancer cells from ferroptosis induced by RSL3 (Brown and Mercurio, 2020).

In ovarian cancer (OVCA), the recurrent resistance to chemotherapeutic drugs is a major clinical challenge. Chemotherapy, particularly employing platinum-based agents and taxanes, remains a cornerstone in the treatment of gynecological malignancies. These agents elicit cell death through distinct mechanisms: platinum drugs via DNA damage and apoptosis (Kleih et al., 2019), and taxanes through microtubule disruption. While up to 80% of patients initially respond to these treatments, the vast majority develop resistance, ultimately leading to treatment failure and disease recurrence. This acquired resistance is a complex phenomenon attributed to many mechanisms, including diminished intracellular cisplatin accumulation, increased drug efflux, alterations in DNA repair pathways, and the activation of anti-apoptotic signaling cascades (Abal et al., 2003). Recently, strong research efforts have been made in discovering new drugs able to either provide an alternative therapeutic approach or to counteract chemotherapy resistance. In this scenario, ferroptosis-based therapies have shown remarkable anti-tumor effects, either used as single agents or in combination with other chemotherapeutic compounds, thus enhancing ovarian cancer cell sensitivity (Zhao et al., 2022; Ruan et al., 2023; Kapper et al., 2024). For instance, the FIN artesunate (ART) alone strongly induces lipid ROS, inhibiting OVCA cells proliferation *in vitro* and reducing tumor growth in mouse models (Greenshields et al., 2017). Inhibition of Stearoyl-CoA 9-desaturase (SCD1), an enzyme responsible for introducing double bonds into saturated fatty acids to produce unsaturated fatty acids, significantly increases long-chain saturated ceramides in membrane phospholipids, thereby promoting ferroptosis in OVCA cell lines and mouse xenograft models. This study also shows that combining SCD1 inhibitors with cisplatin enhances OVCA cells sensitivity to chemotherapy, offering a potential strategy to overcome chemotherapy resistance (Xuan et al., 2022). Additionally, erastin reverses OVCA cell resistance to docetaxel by significantly reducing the expression of ATP Binding Cassette Subfamily B Member 1 (ABCB1), which, when overexpressed, mediates the efflux of docetaxel from OVCA cells and reduces its therapeutic efficacy (Zhou et al., 2019). The interconnection between chemotherapy and ferroptosis is even more complex considering that both cisplatin and paclitaxel have demonstrated the capacity to leverage ferroptosis for enhanced therapeutic benefit. Cisplatin depletes intracellular glutathione (GSH) consequently enhancing lipid peroxidation (Fu et al., 2023). Paclitaxel effectively triggers ferroptosis by suppressing SLC7A11 expression, also leading to GSH depletion, increased oxidative stress, and lipid peroxidation (Lv et al., 2017).

Despite these promising findings, OVCA sensitivity to ferroptosis-based therapeutic approach remains highly variable, and the factors contributing to this variability are still poorly understood (Ruan et al., 2023). Liu et al., showed that OVCA cells chronically exposed to erastin gradually develop resistance to this FIN by constitutively activating NRF2 pathway, leading to the upregulation of cystathionine β-synthase (CBS), which promotes cysteine biosynthesis (Liu et al., 2020). In contrast, Yang et al. demonstrated that the transcriptional coactivator with PDZ-binding motif (TAZ), a Hippo pathway effector, enhances OVCA cells sensitivity to ferroptosis by activating NOX2 (Yang et al., 2020). Furthermore, genome-wide CRISPR-Cas9 suppressor screens and lipidomic profiling by Zou Y et al., revealed that OVCA cell resistance to ferroptosis can result from the downregulation of peroxisomes, leading to the inhibition of PUFA-ePLs synthesis (Zou et al., 2020).

Recently, we observed that OVCA cell sensitivity to erastin is closely associated with the accumulation of intracellular LIP, with ferroptosis resistance occurring when intracellular LIP remains limited, even in the presence of FINs. In agreement, we demonstrated that supplying iron (i.e., ferlixit) in combination with erastin restores ferroptosis sensitivity in resistant OVCA cells (Battaglia et al., 2022). However, the molecular mechanisms preventing intracellular iron overload in response to ferroptotis-inducing stimuli remain poorly understood. In this study, we demonstrate that OVCA cells can resist ferroptosis by exporting iron-rich ferritin via CD63-mediated MVB/EV trafficking. In contrast, specific inhibition of EV biogenesis overcomes ferroptosis resistance in OVCA cells *in vitro*. Overall, our findings provide valuable insights into mechanisms underlying ferroptosis resistance in ovarian cancer and suggest potential therapeutic strategies for enhancing ferroptosis in cancer treatment.

## 2 Materials and methods

### 2.1 Cell lines, cell culture, and reagents

Human epithelial ovarian cancer cell lines HEY, COV318, and PEO4 were purchased from the American Type Culture Collection (ATCC, Rockville, MD, United States). HEY cells were grown in DMEM medium (Sigma-Aldrich, St. Louis, MO, United States), while PEO1 and PEO4 cells were cultured in RPMI 1640 (Sigma-Aldrich, St. Louis, MO, United States), both supplemented with 10% (v/v) fetal bovine serum (FBS) (Invitrogen, San Diego, CA), L-glutamine and 1% (v/v) penicillin and streptomycin (Sigma-Aldrich, St. Louis, MO, United States) in adherent cultures at 37°C in a humidified incubator with 5% CO_2_ atmosphere. All cell lines were tested for *mycoplasma* contaminations and STR profiled for authentication. Erastin, manumycin A (MANU) and GW4869 (GW) were purchased from Sigma Aldrich (Sigma-Aldrich, St. Louis, MO, United States). Each compound was used at the following final concentrations: erastin at 8 µM for 24 h; manumycin A at 500 nM for 24 h; GW4869 at 10 μM for 24 h. Treatments were performed at least three times on independent biological replicates.

### 2.2 Extracellular vesicle isolation and characterization

To isolate EVs, cells were first cultured in the designated media supplemented with 1% penicillin–streptomycin and 10% EV-depleted FBS (Costa-Silva et al., 2015). For the preparation of conditioned culture media, cells were seeded in 150 mm culture dishes containing 20 mL of EV-depleted medium and grown for 72 h until they reached a confluency of ∼90%. Then, the supernatants were immediately submitted to two centrifugation steps (500 × g for 10 min and 3,000 × g for 20 min, both at 10°C) to remove dead cells and large debris. Then, EV samples were isolated by differential ultracentrifugation combined with a sucrose cushion, as previously described (Elzanowska et al., 2022). Briefly, the supernatant was centrifuged at 100,000 × g for 2 h 20 min. The pellet was resuspended in 16 mL of filtered phosphate-buffered saline (PBS; Corning 15,313,581; New York, NY, United States) and added to the top of a 4 mL sucrose solution (D2O containing 1.2 g of protease-free sucrose and 96 mg of Tris base adjusted to pH 7.4). Next, centrifugation at 100,000 × g for 1 h 10 min was performed; then, a 4 mL fraction was collected using an 18G needle and mixed with 16 mL of filtered PBS. After overnight centrifugation at 100,000 × g, the pellet of purified EVs was resuspended in filtered PBS. All used solutions were filtered using 0.22 μm filters. All centrifugation steps were performed at 10°C using 45Ti or 70Ti rotors (Beckman-Coulter, Brea, CA, United States). For each analyzed cancer cell line, at least three independent batches of EVs from separately grown cultures were isolated. To determine particle concentration and size distribution, all EV samples were analyzed by NTA using NanoSight NS300 (Malvern Instruments, Malvern, United Kingdom) with a red laser (638 nm). Samples were diluted with filtered PBS to obtain concentrations within the optimal range for NTA analyses. The videos were recorded using a camera level of 16 and a threshold of 5–7 and further analyzed with NTA software v3.4 (Malvern Instruments, Malvern, United Kingdom). Then, Western blotting was used to assess the presence of EV and non-EV protein markers. Equal protein amounts of EV samples were quantify using Micro BCA Protein assay kit (Thermo Fisher Scientific, Waltham, United States), mixed with 6X Laemmli buffer (Bio-Rad), denatured for 5 min at 95°C, and loaded onto 10%–12% SDS-PAGE and then transferred to nitrocellulose membranes (Sigma-Aldrich, St. Louis, Missouri, United States). After blocking with 5% milk, incubation with primary antibody was performed overnight at 4°C. Membranes were washed with TBS-T (TBS with 0.1% Tween-20) three times for 5 min and then incubated with secondary antibodies for 1 h at RT. Incubation was followed by three additional washes with TBS-T of 5 min each. The antibody against FtH (1:200, sc-376594) was purchased from Santa Cruz Biotechnology, while antibodies against CD63 (1:1000, ab231975) and CD9 (1:1000, ab223052) were obtained from Abcam (Abcam, Cambridge, United Kingdom). The latter was used as exosome marker and references for sample loading.

### 2.3 Transmission electron microscopy (TEM)

OVCA cells (2 × 10^6^ cells/well) were plated in 100 mm culture dishes. Upon treatments, cells were centrifuged, and the relative pellet were fixed for 3 h with 3% glutaraldehyde solution in 0.1 M phosphate buffer (pH 7.4). After washing in PBS for 15′, samples were post-fixed in osmium tetroxide (1%) for 2 h, dehydrated in graded acetone, and then progressively embedded in acetone/resin with final embedment in pure resin (Araldite-Fluka). Ultrathin sections (60–90 nm in thickness) were cut with a diamond knife, collected on copper grids (G300 Cu), and then examined with a Jeol JEM 1400-Plus electron microscope operating at 80 kV.

### 2.4 PI staining analysis

Approximately 1 × 10^6^ cells/well were seeded in 6-well plates overnight followed by the various treatments. Cells were centrifuged and the relative pellets were incubated with (100 μg/mL) iodide propidium (PI) staining in the dark at 37°C for 15 min. Samples were then washed twice with PBS. Fluorescence was analyzed by FACS BD LSRFortessaTM X-20 cytofluorometer (BD Biosciences). A total of 2 × 10^4^ event was acquired for each sample from three independent experiments. Fluorescence was measured using FlowJo software program (Tree Star, Inc.).

### 2.5 Intracellular iron quantification

FerroOrange is a small molecular iron sensing dye containing a fluorescent probe that selectively binds iron ions, allowing for detection of LIP via imaging. For live detection of intracellular iron, OVCA cells were seeded and treated as needed. Then, 1 μmol/L FerroOrange was added and incubated for 30’ at 37°C (Biamonte et al., 2021). Fluorescence intensity was documented using Leica THUNDER Imaging Systems DMi8 (Leica Microsystems S. r.l., Wetzlar, Germany) after 2–4 h of treatments. Each experiment was performed in triplicate.

### 2.6 Mitochondrial ROS and lipid peroxidation analyses

The generation of mitochondrial ROS and lipid peroxidation were measured by flow cytometry using the MitoSOX Red Mitochondrial Superoxide Indicator (Cod. M36008, Thermo Fisher Scientific, Waltham, United States) and BODIPY™ 581/591C11 dye (Cod. D3861, Thermo Fisher Scientific, Waltham, United States), respectively. Upon treatments, single cell suspensions were incubated with 5 µM MitoSOX for 10’ at 37°C or 2.5 µM BODIPY™ 581/591 C11 for 10’ at 37°C; unincorporated dye was removed by washing twice with PBS. Of note, oxidation of BODIPY-C11 resulted in a shift of the fluorescence emission peak from ∼590 nm to ∼510 nm proportional to lipid ROS generation. Specifically, in the reduced state, the excitation and emission maxima of BODIPY™ 581/591C11 is 581/591 nm (PE); after oxidation, the probe shifts the excitation and emission to 488/510 nm (Alexa-Fluor 488). Flow cytometry assays were performed using the BD LSRFortessa™ X-20 (BD Biosciences, San Jose, CA, United States). A minimum of 2 × 10^4^ cells was analyzed per condition. Fluorescence was measured using the FlowJo™ software (Tree Star Inc., Ashland, Oregon, United States). Each experiment was performed in triplicate.

### 2.7 Total protein extraction and Western blotting

Total protein extracts from OVCA cells were obtained using RIPA buffer containing 1 M Tris HCl, Triton X-100, 3 M NaCl, 0.5 M EDTA, 10% SDS supplemented with cOmplete™ Protease Inhibitor Cocktail provided in EASYpacks (Roche Diagnostics, Mannheim, Germany), as previously reported (Guglielmelli et al., 2010; Oliveira et al., 2018; Di Sanzo et al., 2022). Briefly, cells were lysed in ice-cold RIPA buffer and, after removal of the cell insoluble fragments through centrifugation at 12.000 g for 30 min at 4°C, protein content was quantified by Bio-Rad Protein Assay Dye according to manufacturer’s instructions (Bio-Rad Laboratories, Hercules, California, United States). Each protein sample (40–50 µg) was separated by using 10%–15% SDS-PAGE and then transferred to nitrocellulose membranes (Sigma-Aldrich, St. Louis, Missouri, United States). After blocking with 5% milk, incubation with primary antibody was performed overnight at 4°C. The antibody against FtH (1:200, sc-376594) was purchased from Santa Cruz Biotechnology (Cruz Biotechnology, Dallas, Texas, United States); antibodies against CD63 (1:1000, ab231975), FPN (1:500, ab235166), Prominin2 (PROM2) (1:500, ab74977), were purchased from Abcam (Abcam, Cambridge, United Kingdom), while antibody against CD71 (1:1,000, #13208) was obtained from Cell Signaling Technology (Danvers, Massachusetts, United States). After incubation with peroxidase-conjugated secondary antibodies (Peroxidase AffiniPure Sheep Anti-Mouse IgG, 1:10,000; Peroxidase AffiniPure Donkey Anti-Rabbit IgG, 1:10000; Peroxidase AffiniPure Donkey Anti-Goat IgG, 1:10,000; Jackson ImmunoResearch Europe Ltd., Cambridge, United Kingdom) for 1 h at room temperature, signals were detected using chemiluminescence reagents (ECL Western blotting detection system, Santa Cruz Biotechnology, Dallas, Texas) and acquired by Uvitec Alliance Mini HD9 (Uvitec Cambridge, United Kingdom). To calculate the relative expression of specific protein, a goat polyclonal anti-γ-Tubulin antibody (γ-TUB, 1:3000; sc-17787; Santa Cruz Biotechnology) and a mouse monoclonal IgG GAPDH HRP (1:3000; sc-47724) serve as references for sample loading.

### 2.8 *FtH* and *CD63* transient knockdown

OVCA cells were transfected using Lipofectamine™ 3,000 Transfection Reagent (Thermo Fisher Scientific, Waltham, MA, United States) as previously described (Di Sanzo et al., 2016; Chirillo et al., 2020). *FtH* and *CD63* siRNA were purchased from Thermo Fisher Scientific. To ensure an optimal control, cells were further transfected with Silencer™ Select Negative Control siRNA (ctrl) (Thermo Fisher Scientific, Waltham, MA, United States). The transfection efficiency was evaluated by using qRT-PCR.

### 2.9 RNA isolation and comparative qRT-PCR analysis

Total RNA extraction was obtained through the Trizol RNA isolation method (Life Technologies, Carlsbad, California, United States) as previously described (Zolea et al., 2016; Biamonte et al., 2017; Battaglia et al., 2023a; Sacco et al., 2023). All samples were DNase treated (Thermo Fisher Scientific, Waltham, Massachusetts, United States) and purity/integrity check was performed spectroscopically before use. Then, 1 µg of total RNA was retrotranscribed using Applied Biosystems™ High-Capacity cDNA Reverse Transcription Kit (Thermo Fisher Scientific, Waltham, Massachusetts, United States). qRT-PCR was performed using the SYBR™ Green qPCR Master Mix (Thermo Fisher Scientific, Waltham, Massachusetts, United States). Analysis was performed on Applied Biosystems™ QuantStudio™ 3 (Thermo Fisher Scientific). The relative mRNA expression level of FtH and CD63 were calculated through the 2^−ΔΔCT^ method and glyceraldehyde 3-phosphate dehydrogenase (GAPDH) was used as the housekeeping gene. Each experiment was performed in triplicate. Primers used for qRT-PCR are as follows: FtH (FW: 5′-TTGACCGAGATGATGTGGCT-3′, REV: 5′-CCAGTTTGTGCAGTTCCAGT-3′); CD63 (FW: 5′-TCCTCTTCCTGGTGGCTTTT-3′, REV: 5′-CCACAGCCCACAGTAACATT-3′); GADPH (FW: 5′-CAAATTCCATGGCACCGTCA-3′, REV: 5′-GGCAGAGATGATGACCCTTT-3′).

### 2.10 Statistical analysis

Overall data are represented as mean ± standard deviation (SD) of at least three biological replicates. When appropriate, data were analyzed by performing a simple comparison between two groups using Student’s t-test. A *p*-value <0.05 was considered statistically significant.

## 3 Results

### 3.1 Erastin resistance is associated with CD63-dependent MVB formation in OVCA cells

To investigate the mechanisms that promote ferroptosis resistance in OVCA, we leveraged our recent finding that treatment with the canonical ferroptosis inducer erastin (8 µM, 8 h) is associated with ferritinophagy, LIP accumulation, and approximatively 25% mortality in HEY cells. In contrast, COV318 and PEO4 cells, which exhibit resistance to erastin treatment, show upregulation of FtH without alteration in the LIP. Notably, administration of Fe^3+^ (i.e., ferlixit) restores erastin sensitivity in COV318 and PEO4 cells (Battaglia et al., 2022).

**FIGURE 1:**
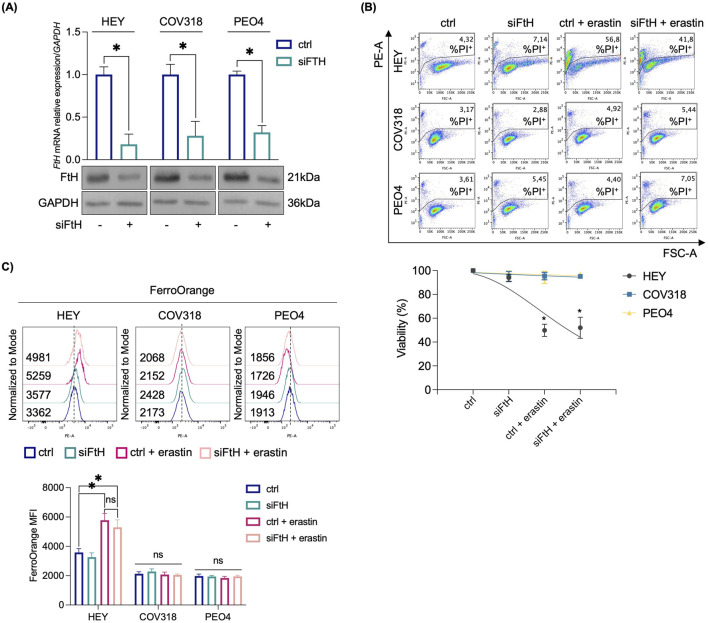
Erastin-mediated ferroptosis in OVCA cells is independent from *FtH* knockdown. **(A)** qRT-PCR and Western blot analyses of FtH in HEY, COV318 and PEO4 cells (*siFTH* vs*.* ctrl). GAPDH was used as a normalization control for mRNA and protein quantification. **(B)** PI flow cytometric analysis and relative histogram of OVCA cells (*siFtH* vs*.* ctrl) upon treatment with 8 μM erastin; % of dead cells (PI positive) are reported in each dot plot. **(C)** Flow cytometry analysis and relative histogram of LIP content with FerroOrange dye in OVCA cells (*siFtH* vs*.* ctrl) treated or not with 8 μM erastin. All the experiments were carried out in triplicate. Histograms are presented as mean ± SD. *p-*value: *<0.05. ns: not significant.

Here, once replicated PI flow cytometry assays of HEY, COV318, and PEO4 cells treated with growing concentrations of erastin (2 µM, 4 µM, 8 µM) at two-time points (8 h and 24 h) (Supplementary Figure S1), we explored the hypothesis that, to avoid erastin-mediated cytotoxicity, COV318 and PEO4 cells activate protective mechanisms that limit LIP accumulation. First, to assess whether loss of the FtH-mediated iron storage contributes to ferroptosis sensitivity in resistant cells, we generated *FtH*-deficient COV318 and PEO4 cells. We analyzed their response to treatment with 8 µM erastin for 24 h. As shown in Figures 1A, B, siRNA-mediated knockdown of *FtH* (70% reduction) was insufficient to sensitize COV318 and PEO4 to erastin-induced ferroptosis (PI^+^ COV318^ctrl^: 3.17%, PI^+^ COV318^siFtH^: 2.88%, PI^+^ COV318^ctrl/era^: 4.92%; PI^+^ COV318^siFtH/era^: 5.44%) (PI^+^ PEO4^ctrl^: 3.61%, PI^+^ PEO4^siFtH^: 5.45%, PI^+^ PEO4^ctrl/era^: 4.40%; PI^+^ PEO4^siFtH/era^: 7.05%). Indeed, no alteration in the LIP was detected in response to *FtH* knockdown (Figure 1C).

**FIGURE 2:**
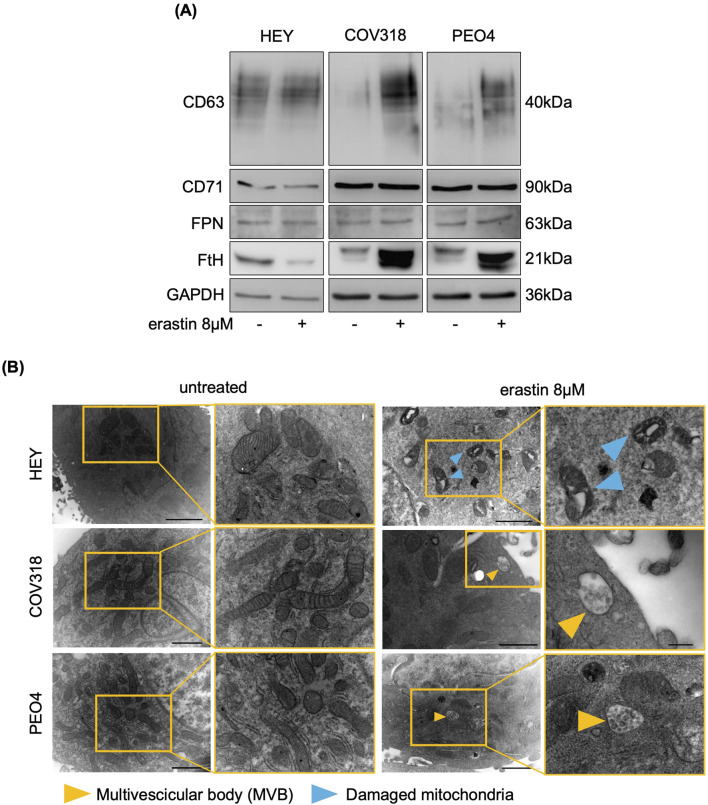
Erastin induces the accumulation of CD63-related MVBs in resistant OVCA cells. **(A)** Western blot analysis of FtH, FPN, CD71 and CD63 in OVCA cells upon administration of 8 μM erastin. GAPDH serves as loading control. **(B)** Representative images of morphological and ultrastructural features detected by TEM in HEY, COV318 and PEO4 cells treated with or without 8 μM erastin. Yellow arrows, multivescicular body (MVB); blue arrows, damaged mitochondria. All the experiments were carried out in triplicate.

**FIGURE 3:**
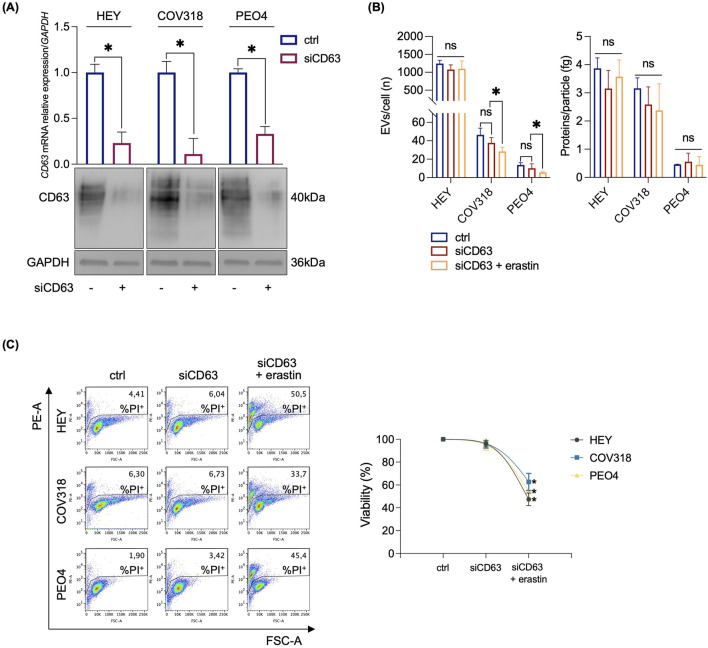
CD63 knockdown sensitizes resistant OVCA cells to erastin mediated ferroptosis. **(A)** qRT-PCR and Western blot analyses of CD63 in HEY, COV318 and PEO4 cells (*siCD63* vs*.* ctrl). GAPDH was used as a normalization control for mRNA and protein quantification. **(B)** Histograms showing the number of EVs/cell and the amount of protein/particle (fg) secreted from HEY, COV318 and PEO4 cells following CD63 silencing and treated with 8 μM erastin. **(C)** PI flow cytometric analysis and relative histograms of OVCA cells (*siCD63* vs*.* ctrl) upon treatment with 8 μM erastin; % of dead cells (PI positive) are reported in each dot plot. Each experiment was performed in triplicate. Histograms are presented as mean ± SD. *p-*value: *<0.05. ns: not significant.

**FIGURE 4:**
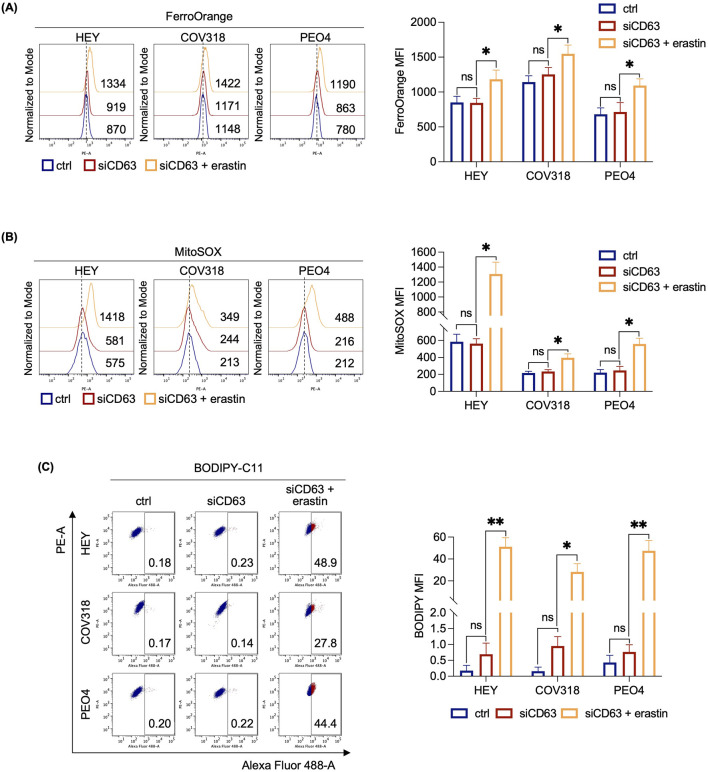
CD63 knockdown induces LIP accumulation, mitochondrial dysfunction and lipid peroxidation in OVCA cells resistant to erastin. Flow cytometry analysis of LIP content **(A)**, mitochondrial ROS levels **(B)** and lipid peroxidation **(C)** quantified by using FerroOrange, MitoSOX and BODIPY-C11 reagents, respectively, in OVCA cells (siCD63 vs*.* ctrl) after the administration of 8 μM erastin. Each experiment was performed in triplicate. Histograms are presented as mean ± SD. *p-*value: *<0.05; **<0.01. ns: not significant.

Next, we assessed any possibile variation in either iron intake or export by quantifying the expression of CD71 and FPN in HEY, COV318, and PEO4 cells following erastin treatment. As shown in Figure 2A, no significant changes in CD71 or FPN expression were detected. An alternative explanation for our hypothesis was that OVCA cells prevent LIP accumulation by promoting its expulsion via the EV pathway. Recently, expulsion of iron-loaded ferritin via CD63-mediated or prominin2-mediated MVB/EV trafficking was described as key mechanisms for limiting LIP levels within cancer cells (Brown et al., 2019; Yanatori et al., 2021). Based on these recent findings, we quantified CD63 and prominin2 expression in cell lysates by WB. We found that CD63 was significantly upregulated in response to erastin treatment in resistant OVCA cells (COV318 and PEO4), but not in sensitive HEY cells (Figure 2A). The immunoblots revealed a laddered banding pattern for CD63 at ∼30–60 kDa, with a prominent band around 40 kDa indicative of differential glycosylation (Yanatori et al., 2021). In contrast, no significant change in prominin2 was observed after erastin treatment (Supplementary Figure S2). In agreement, TEM analysis revealed a marked increase in MVBs, with distinct intraluminal vesicles (ILVs), in COV318 and PEO4 within 4 h of erastin treatment compared to control cells. This effect was not observed in HEY cells (Figure 2B). Notably, CD63 knockdown reduced EVs release in COV318 and PEO4 cells treated with erastin, without affecting those released by HEY cells (Figures 3A, B). In parallel, it enhanced sensitivity of COV318 and PEO4 cells to erastin-induced cell death (PI^+^ COV318^ctrl^: 6.30%, PI^+^ COV318^siCD63^: 6.73%, PI^+^ COV318^siCD63/era^: 33.7%) (PI^+^ PEO4^ctrl^: 1.90%, PI^+^ PEO4^siCD63^: 3.42%, PI^+^ PEO4^siCD63/era^: 45.4%) (Figure 3C), along with a significant increase in intracellular LIP accumulation (Figure 4A), mitoROS production (Figure 4B), and lipid peroxidation (Figure 4C).

### 3.2 Erastin resistance is facilitated by MVB/exosome ferritin expulsion in OVCA cells

**FIGURE 5:**
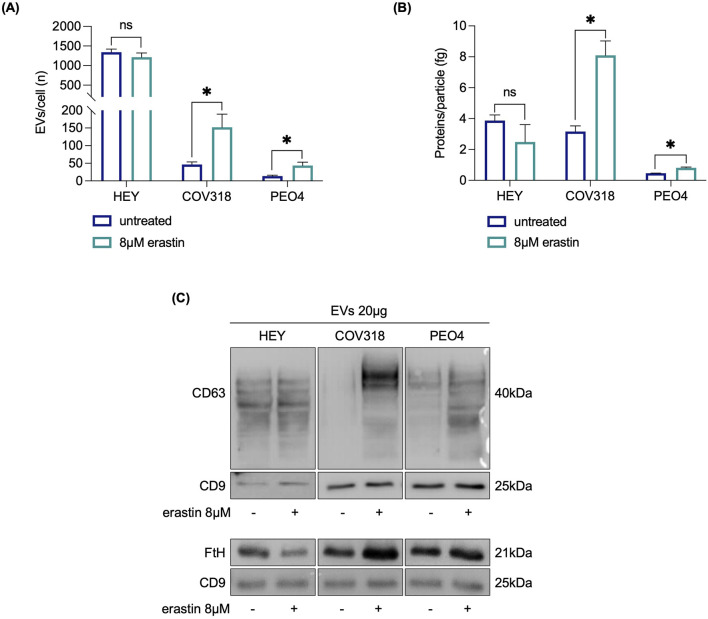
FtH protein expression increases in EVs secreted by OVCA cells resistant to erastin. Histograms showing the number of EVs/cell **(A)** and the amount of protein/particle (fg) **(B)** secreted from HEY, COV318 and PEO4 cells following the administration of 8 μM erastin. **(C)** CD63 and FtH protein expression were detected by Western blot in HEY, COV318 and PEO4 cells after treatment with erastin. CD9 was used as exosome marker and normalization control. All data represent the mean of three indipendent experiments. qRT-PCR are presented as mean ± SD and the statistical significance was expressed as *p-*value: *<0.05. ns: not significant.

Based on the observation that erastin induces CD63 expression and promotes MVBs formation in ferroptosis-resistant COV318 and PEO4 cells, we next evaluated the effect of erastin on EVs formation. HEY, COV318, and PEO4 cells were cultured for 72 h in media containing bovine EV-depleted FBS, and then treated with erastin. EVs were harvested from the cellular supernatants by ultracentrifugation and analyzed using NTA. As shown in Figures 5A, B, the number of EVs/cell secreted from COV318 and PEO4 cells increased approximately 3-fold upon erastin treatment (COV318: 151.95 vs*.* 46.38; PEO4: 43.49 vs*.* 13.78) (*p* < 0.05) as well as the amount of protein/particle (COV318: 8.09 vs*.* 3.16; PEO4: 0.81 vs*.* 0.46) (*p* < 0.05). Immunoblot analysis revealed a remarkable increase in CD63 expression in EVs isolated from erastin-treated COV318 and PEO4 cells compared to the untreated counterparts, confirming that EVs were generated by CD63-dependent MVB formation (Figure 5C). In contrast, no change in CD63 expression was detected in EVs from erastin-treated HEY cells, which also showed no alteration in the number of EVs/cell (Figures 5A–C). Importantly, WB analysis of FtH in the EVs fraction showed a significant and pronounced increase exclusively in EVs isolated from erastin-treated COV318 and PEO4 cells (Figure 5C). These findings suggest that, in OVCA-resistant cells, erastin is associated with CD63-mediated MVB/EV generation, leading to expulsion of ferritin and contributing to ferroptosis resistance.

### 3.3 Inhibition of EV biogenesis prevents iron-rich ferritin expulsion and resensitizes OVCA cells to erastin

**FIGURE 6:**
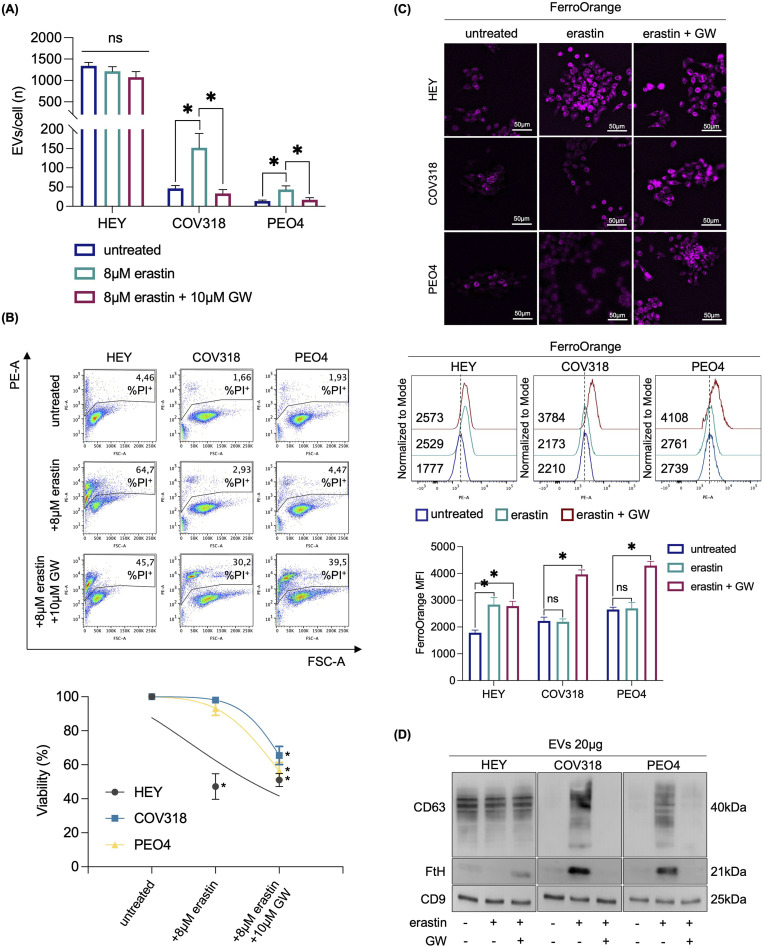
Inhibition of EVs biogenesis prevents iron-rich ferritin expulsion and repristinates erastin sensitivity in OVCA cells. **(A)** Histograms showing the number of EVs/cell secreted from HEY, COV318 and PEO4 cells following the administration of 8 μM erastin alone or in combo with 10 μM GW. **(B)** PI flow cytometry assay and relative histograms of HEY, COV318 and PEO4 cells treated with erastin and in combination with 10 μM GW % of dead cells (PI positive) are reported in each dot plot. **(C)** Fluorescence microscopy analysis, flow cytometry assay and relative histograms of LIP content with FerroOrange dye in OVCA cells upon erastin administration with or without 10 μM GW. **(D)** CD63 and FtH protein expression were detected by Western blot in HEY, COV318 and PEO4 cells after treatment with erastin alone or in combo with GW. CD9 was used as exosome marker and normalization control. Each experiment was performed in triplicate. Histograms are reported as mean ± SD and the statistical significance was expressed as *p-*value: *<0.05. ns: not significant.

**FIGURE 7:**
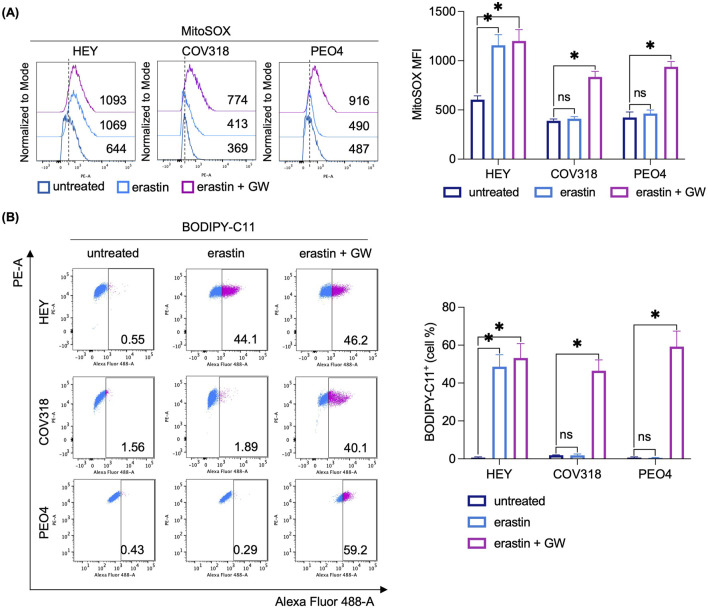
Inhibition of EVs biogenesis induces mitochondrial dysfunction and lipid peroxidation in OVCA cells resistant to erastin. Flow cytometry analysis and relative histograms of mitochondrial ROS levels **(A)** and lipid peroxidation **(B)** quantified by using MitoSOX and BODIPY-C11 reagents, respectively, in OVCA cells following the single treatment with erastin and its combination with GW. Each experiment was performed in triplicate. Histograms are reported as mean ± SD and the statistical significance was expressed as *p-*value: *<0.05.

To determine whether CD63^+^EV-mediated ferritin release is essential for limiting LIP accumulation and promoting erastin resistance in COV318 and PEO4 cells, we inhibited EV biogenesis and secretion using the sphingomyelinase inhibitor GW4869 (GW). Briefly, HEY, COV318, and PEO4 cells were co-treated with erastin and 10 μM GW for 8 h. Once we verified the reduction in the number of EV/cell (Figure 6A), and excluded any possibile cytotoxic effect of GW alone (Supplementary Figure S3A), we found that GW significantly increased the sensitivity of COV318 and PEO4 cells to erastin (PI^+^ COV318^untr^: 1.66%, PI^+^ COV318^era^: 2.93%, PI^+^ COV318^era/GW^: 30.2%; *p* < 0.01) (PI^+^ PEO4^untr^: 1.93%, PI^+^ PEO4^era^: 4.47%, PI^+^ PEO4^era/GW^: 39.5%; *p* < 0.01) (Figure 6B). Diversely, the use of manumycin A, an ESCRT-dependent EVs biogenesis inhibitor, showed no effects on EV number nor ferroptosis sensitivity in COV318 and PEO4 cells (Supplementary Figures S3B, C). Using FerroOrange live staining, we observed that GW combined with erastin increased the concentration of intracellular free iron in COV318 and PEO4 cells, with the most pronounced effect occurring 2 h post-erastin-treatment (Figure 6C). In agreement, the amounts of CD63 and FtH in EVs were significantly reduced (Figure 6D). As evidence of ferroptosis induction in COV318 and PEO4 cells, MitoSOX and BODIPY-C11 flow cytometry assays revealed that GW combined with erastin causes a ∼ 2-fold increase in mitoROS (MFI MitoSOX, COV318^untr^: 369, COV318^era^: 413, COV318^era/GW^: 774; *p* < 0.05) (MFI MitoSOX, PEO4^untr^: 487, PEO4^era^: 490, PEO4^era/GW^: 916; *p* < 0.05) and a significant accumulation of lipid peroxides (BODIPY-C11, COV318^untr^: 1.56%, COV318^era^: 1.89%, COV318^era/GW^: 40.1%; *p* < 0.01) (BODIPY-C11, PEO4^untr^: 0.43%, PEO4^era^: 0.29%, PEO4^era/GW^: 59.2%; *p* < 0.01) (Figure 7A, B). No significant changes in the biochemical markers of ferroptosis were detected in HEY cells upon co-treatment with erastin and GW. Overall, these results indicate that CD63^+^ EV-mediated expulsion of iron-rich ferritin is a protective mechanism against ferroptotic cell death and that inhibition of EV biogenesis can represent a potential therapeutic strategy to overcome ferroptosis resistance in OVCA.

## 4 Discussion

Ferroptosis has emerged as a potent form of non-apoptotic cell death, serving as a strong “Achille’s heel” in cancer cells and thus a promising target for novel therapeutic strategies against OVCA (Li et al., 2021). However, inherited and acquired resistance represent a bottleneck in the application of FINs in the treatment of this malignancy (Wang et al., 2024). Therefore, understanding the molecular mechanisms underlying ferroptosis resistance in OVCA is an urgent priority. Recently, we and others have suggested that ferroptosis resistance in certain OVCA cells might be due to dysregulation of iron metabolism, which ultimately impacts the intracellular accumulation of free and redox-active iron (Battaglia et al., 2022). Specifically, we have previously shown that treatment with the canonical FIN, erastin, efficiently kills HEY cells but not COV318 and PEO4 OVCA cells. While erastin administration in HEY cells is associated with ferritinophagy and LIP accumulation, LIP remains unaltered in COV318 and PEO4 cells. Notably, the use of iron compounds (e.g., ferlixit) in combination with erastin restores ferroptosis sensitivity in these resistant OVCA cell lines (Battaglia et al., 2022). The mechanisms limiting the intracellular LIP accumulation in COV318 and PEO4 cells, though, remained unexplored.

In this study, we demonstrate that the mechanisms counteracting LIP accumulation in erastin-resistant OVCA cells are not associated with reduced CD71-mediated iron uptake nor increased iron export via FPN. Rather, COV318 and PEO4 cells upregulate the expression of FtH, which in turn is secreted as iron-rich ferritin via CD63^+^ EVs. CD63 is a member of the tetraspanin superfamily; it is localized to the ILVs encapsulated within the MVBs and is involved in EVs secretion (Toribio and Yáñez-Mó, 2022). EVs are membrane vesicles of endocytic origin whose biogenesis is mediated by two distinct pathways, namely, ESCRT-dependent and ESCRT-independent. ESCRT is a multi-proteins complex that interacts with ubiquitylated cargos and promotes the formation of ILVs. The ESCRT pathway is associated with RAS, a small GTPase able to orchestrate different cellular processes including cell proliferation, differentiation, adhesion, and migration. To function properly, RAS must be linked to a lipid chain through the activity of specific enzymes called farnesyltransferases. The ESCRT-independent pathway is, instead, orchestrated by neutral sphingomyelinases, a family of enzymes able to convert sphingomyelin, present inside lipid raft, in ceramide. The resulting ceramides form large microdomains inducing the budding and the maturation of ILVs into MVBs (Catalano and O’Driscoll, 2019). EVs play key roles in intercellular communication, transferring molecular cargo, such as proteins, DNAs, mRNAs and microRNAs, between cancer cells and their microenvironment (Maia et al., 2018). Recently, EVs have been also involved in the regulation of intracellular iron homeostasis through the mobilization of iron-rich ferritin outside of cells (Brown et al., 2019; Yanatori et al., 2021). Yanatori I. et al., found that, when iron levels increase, iron regulatory proteins IRP1 and IRP2 bind to the iron-responsive element (IRE) in the 5′untranslated region (UTR) of CD63 mRNA, thereby stimulating its post-transcriptional overexpression. CD63 overexpression, then, promotes the release of iron-rich ferritin via EVs to prevent excessive iron loading (Yanatori et al., 2021). In addition, Brown C.W. et al. Reported that treatment with the ferroptosis inducer RSL3 enhances the expression of PROM2, a pentaspanin protein involved in lipid dynamics and membrane structure, which promotes the formation of MVBs and EVs responsible of transporting iron-rich ferritin out of mammary epithelial and breast cancer cells. This phenomenon limits intracellular LIP levels and causes ferroptosis resistance (Brown et al., 2019).

Here, we demonstrate for the first time that the limited LIP accumulation, which restrains ferroptosis execution in COV318 and PEO4 OVCA cells treated with erastin, can be explained by the export of iron-rich ferritin via CD63^+^ EVs. Mechanistically, in COV318- and PEO4-resistant cells but not in HEY-sensitive cells, erastin treatment is accompanied by the upregulation of CD63 and FtH. As both proteins are post-transcriptionally upregulated by IRE-IRP system under high iron overload conditions (Yanatori et al., 2021), we hypothesized that erastin treatment increases cytosolic iron in COV318 and PEO4 cells but that these cells activate two distinct iron-buffering mechanisms, namely, the iron storage via FtH1 and the iron secretion via CD63^+^ vesicles, to mitigate free iron availability. Subsequently, iron-rich ferritin is incorporated into MVBs and exported outside of the cells via CD63^+^ EVs. No variation in intracellular PROM2 expression was instead observed. This result strengthens the hypothesis that the observed phenomenon was a specific response against iron overload. Although both CD63 and PROM2 are implicated in mitigating ferroptosis through EV-mediated ferritin expulsion in various cancer models, indeed, their regulation is different. Upregulation of PROM2 is observed in response to general ferroptotic stress (e.g., GPX4 depletion or dysfunction) (Brown et al., 2019); that of CD63 is specifically triggered under iron overload conditions (Yanatori et al., 2021).

To further demonstrate the central role of EV-mediated FtH expulsion in COV318 and PEO4 cells, we assessed the effects of EVs inhibition on erastin sensitivity by using GW4869 and manumycin A, an ESCRT-independent and ESCRT-dependent EVs inhibitors, respectively (Catalano and O’Driscoll, 2019). Our results showed that GW4869 substantially bypasses ferroptosis resistance of COV318 and PEO4 cells by reducing CD63 and FtH expression, restoring intracellular LIP accumulation, mitoROS production, and lipid peroxidation. Similarly, CD63 knockdown reduced EVs release and restored intracellular free iron accumulation and erastin cytotoxicity in COV318 and PEO4 cells. Conversely, manumycin A had no effects on EV number or ferroptosis sensitivity. These results suggest that iron-rich ferritin release via CD63^+^ EVs in response to erastin in COV318 and PEO4 cells can be mainly mediated by the ESCRT-independent pathway. However, at present, we cannot exclude that GW4869 treatment or CD63 knockdown can affect ferroptosis sensitivity through the modulation of other cargos, in particular exosomal nucleic acids, such as non-coding RNAs (ncRNAs), which have demonstrated regulatory roles in ferroptosis across diverse disease models (Liu et al., 2024). Indeed, current research investigating the impact of EVs on ferroptosis primarily characterize EVs derived from non-cancer cells within the tumor microenvironment (TME). The mechanisms governing EV-mediated ferroptosis regulation via autocrine pathways instead warrant further investigation. We also speculated whether FtH1 knockdown could restore ferroptosis sensitivity in COV318 and PEO4 cells. Our results demonstrated that FtH1 silencing alone is insufficient to promote erastin responsiveness. This lack of sensitization can be attributable to two interconnected factors. First, FtH1 silencing alone does not affect the iron overload-dependent expression of CD63, which in turn can continue to promote the release of iron-rich ferritin via EVs. Second, and perhaps concurrently, FtH1 silencing can paradoxically exacerbate intracellular free iron levels, which in turn could further stimulate CD63 protein expression and CD63-mediated ferritin expulsion, thereby reinforcing the resistance phenotype.

Concerning HEY sensitive cells, we found that, upon erastin-mediated ferritinophagy and LIP accumulation, these cells fail to activate compensatory iron buffering mechanisms, ultimately succumbing to ferroptosis. HEY cells, although exhibiting a higher baseline EVs output compared to both COV318 and PEO4 cells, maintain the number of EVs released unaffected upon either erastin, or GW4869, or CD63 knockdown. Only manumycin A was found to be able to suppress the number of EVs released by HEY cells. This difference in the regulation of EVs pathway homeostasis is in line with the concept of exosome heterogeneity, encompassing variations in quantity, characteristics, and functional properties across diverse cell types (Willms et al., 2016; Li et al., 2020).

In summary, our findings reveal a previously unrecognized mechanism that contributes to variation in ferroptosis sensitivity in OVCA. Specifically, we show that the dynamic CD63^+^ EV-mediated export of iron-rich ferritin serves as a homeostatic mechanism to restrict iron accumulation, thereby preventing ferroptotic cell death under stress conditions. Furthermore, we demonstrate that targeting EV biogenesis and secretion could have therapeutic implications for overcoming ferroptosis resistance in OVCA cells.

## Data Availability

The original contributions presented in the study are included in the article/Supplementary Material, further inquiries can be directed to the corresponding author.
