# Supplementary material for: Expulsion of iron-rich ferritin via CD63-mediated exosome drives ferroptosis resistance in ovarian cancer cells
Source: Front Cell Dev Biol. 2025 Mar 10;13:1532097. doi: 10.3389/fcell.2025.1532097 (PMC11962263; doi:10.3389/fcell.2025.1532097)
Supplement: Supplementary file 1 [file DataSheet1.pdf]

frontiers

Supplementary Material

Ovarian cancer cells resist ferroptosis through CD63-mediated exosome expulsion of iron-rich ferritin

Anna Martina Battaglia[1], Alessandro Sacco[1], Emanuele Giorgio[1], Lavinia Petriaggi[1], Julia Elzanowska[2], Ana Rita Cruz[2], Luis Rocha[2], Catarina Esteves Pereira[2], Maria Carolina Strano Moraes[2], Luca Palazzo[3], Claudia De Vitis[4], Bruno Costa-Silva[2†], Flavia Biamonte[1†*]

*** Correspondence:** Flavia Biamonte: flavia.biamonte@unicz.it

Supplementary Figures

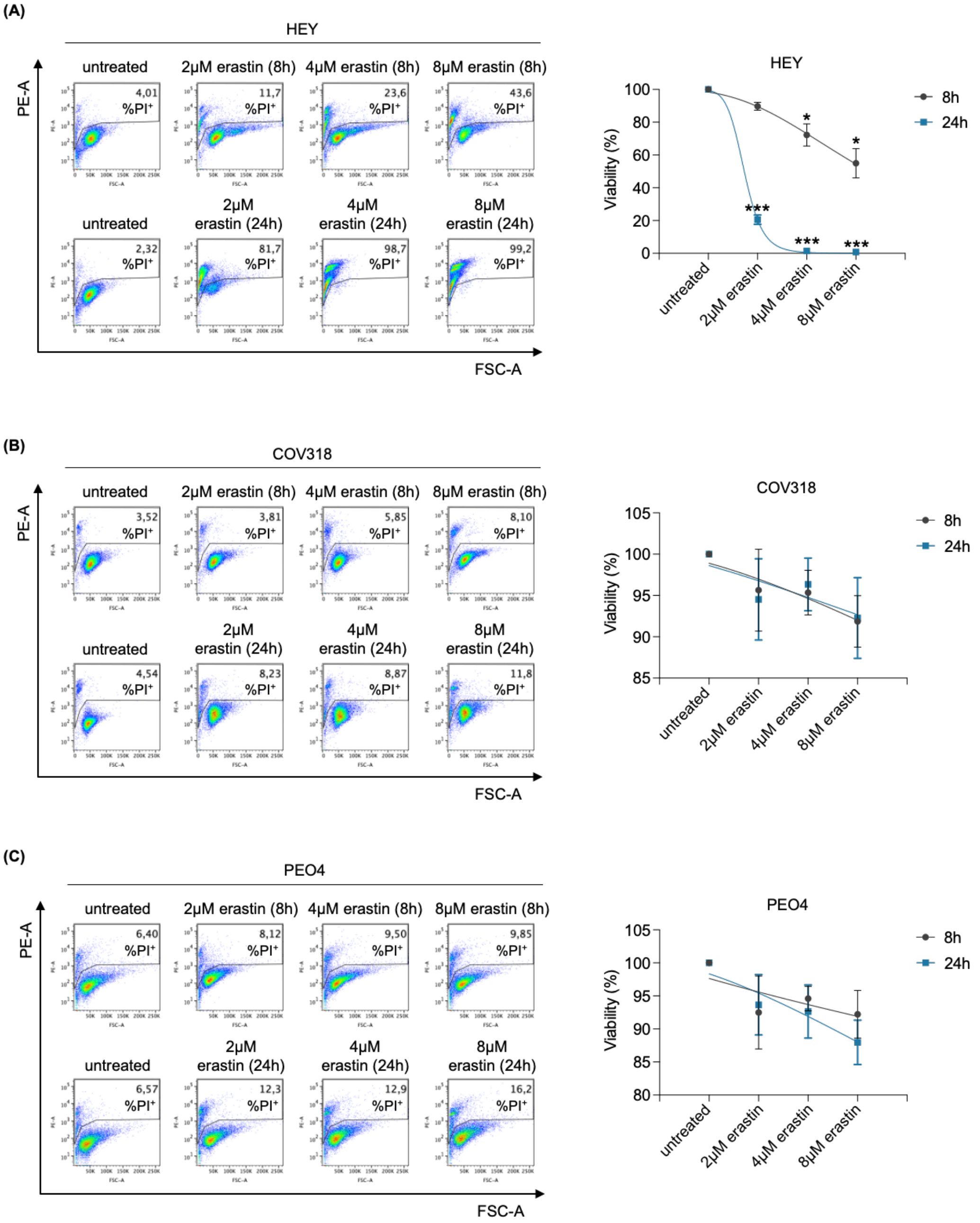

Supplementary Figure 1. Erastin sensitivity is cell type-dependent in OVCA cells. Dose-response and time course analysis of PI flow cytometry assay and relative histograms of HEY, COV318 and PEO4 cells treated with 2µM, 4µM, 8µM erastin for 8h and 24h. % of dead cells (PI positive) are reported in each dot plot. Data represent the mean of three independent experiments. Histograms are reported as mean ± SD. *p*-value: *< 0.05; ***<0.001.

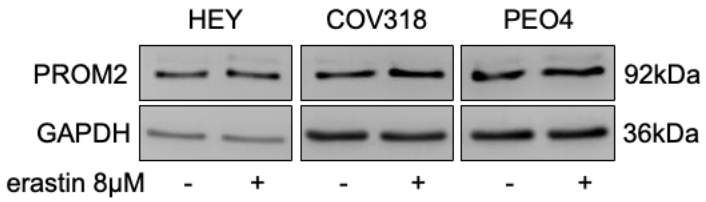

Supplementary Figure 2. PROM2 protein expression remains unaltered upon erastin treatment in OVCA cells. Western blot analysis of PROM2 in OVCA cells upon administration of 8µM erastin. γ-TUB serves as loading control. The experiment was carried out in triplicate.

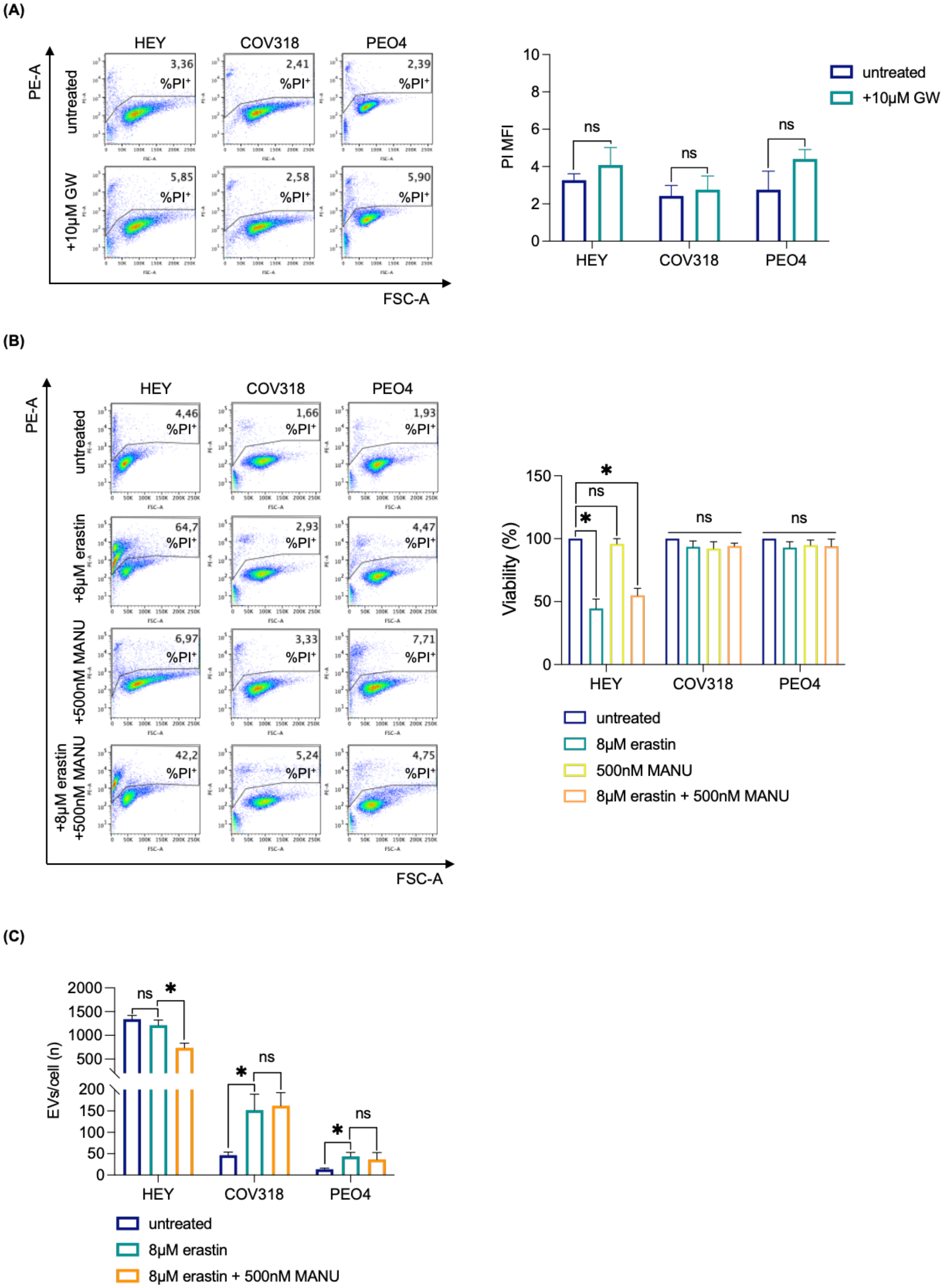

Supplementary Figure 3. (A) PI flow cytometry assay and relative histograms of HEY, COV318 and PEO4 cells treated with 10µM GW. % of dead cells (PI positive) are reported in each dot plot. **(B)** PI flow cytometry assay and relative histograms of HEY, COV318 and PEO4 cells treated with 8µM

erastin and 500nM MANU alone or in combination. % of dead cells (PI positive) are reported in each dot plot. **(C)** Histograms showing the number of EVs/cell secreted from HEY, COV318 and PEO4 cells treated with 8μM erastin alone or in combination with 500nM MANU. Data represent the mean of three independent experiments. Histograms are reported as mean ± SD. *p*-value: *< 0.05. ns: not significant.
